# Multi-Scale Simulation of Injection Molding Process with Micro–Features Replication: Relevance of Rheological Behaviour and Crystallization

**DOI:** 10.3390/polym13193236

**Published:** 2021-09-24

**Authors:** Sara Liparoti, Vito Speranza, Roberto Pantani, Giuseppe Titomanlio

**Affiliations:** 1 Department of Industrial Engineering, University of Salerno, Via Giovanni Paolo II, 132, 84084 Fisciano, SA, Italy; sliparoti@unisa.it (S.L.); rpantani@unisa.it (R.P.); 2 Institute of Polymers, Composites and Biomaterials (IPCB), The National Research Council, Via Previati 1/C, 23900 Lecco, LC, Italy; gtitomanlio@unisa.it

**Keywords:** micro injection molding, flow induced crystallization, mold temperature

## Abstract

The possibility of tailoring key surface properties through the injection molding process makes it intriguing from the perspective of sustainability enhancement. The surface properties depend on the replication accuracy of micro and nanostructures on moldings; such an accuracy is enhanced with cavity temperature. The simulation of the injection molding process is very challenging in the presence of micro and nanostructures on the cavity surface; this does not allow for the neglect of phenomena generally considered not to influence the overall process. In this paper, a multiscale approach was proposed: in the first step, the simulation of the overall process was conducted without considering the presence of the microstructure; in the second step the outputs of the first step were used as an input to simulate the replication of the microfeature. To this purpose, a lubrication approximation was adopted, and the contribution of the trapped air, which slows down the polymer advancement, was accounted for. A modification of the viscosity equation was also proposed to describe the rheological behavior of isotactic polypropylene at very low temperatures. Concerning the microcavity filling simulation, the modification of the viscosity description at low temperatures consistently describes the process, in terms of polymer solidification. Concerning the replication accuracy, it increases with the cavity surface temperature, consistently with the experimental observations.

## 1. Introduction

The injection molding process is one of the most widespread processes for plastic part production. High dimensional accuracy and the possibility to obtain parts with very complex shapes are the more relevant advantages of this process which make it very useful in many fields, from automotive to biomedical, including many others [1,2,3]. Nowadays, the necessity of tailoring surface properties on the moldings has arisen, and several approaches, based on the creation of micro and nanofeatures on the molding surface, have been proposed [4,5,6,7,8,9].

More recently, the production of micro and nanopatterned surfaces in a single-step injection molding process has been proposed [10,11,12]. In this case, a patterned mold insert, previously obtained through lithography-based approaches, was located on the cavity surface, and the replication was assured through the optimization of process parameters, especially injection pressure and cavity temperature [13]. The fact that a one-shot approach is sufficient to obtain a patterned object, made of just one single material, makes injection molding very intriguing from the point of view of the sustainability of both material and process. The possibility of modulating the cavity surface temperature during the process makes it possible to obtain micro and nanostructured surfaces with high geometrical accuracy [13,14].

Many efforts have been devoted to simulating the replication of micro-structured surfaces during injection molding. Some authors have proposed a multi-scale approach by commercial software for injection molding simulation to predict the replication in terms of volume of micro-channel filled during the process. The critical aspect is the appropriate selection of the mesh: a two-scale meshing must be adopted to ensure sufficient precision in the simulation where micro-features are present [11,15]. In other cases, two-step simulations were conducted, adopting the output of the injection molding simulation, without micro-features, as the input to the simulation of the flow into the micro-channels [16,17]. The two-step simulation is based on the hypothesis that the replication can be considered equivalent to two consecutive processes: during the first step, the polymer fills the macro-cavity, without noticing the presence of micro-features; during the second step, the polymer enters the micro-channels due to the pressure profile evolution identified during the macro-cavity filling [18]. During such a pressure-driven flow into the micro-cavity, the local polymer pressure is adjusted for capillary pressure, viscous pressure drop, and pressure of the air held inside the micro-cavity [12]. Generally, these approaches include the heat transfer coefficient, wall slip, and solidification, key factors in micro-filling [19]. The selection of an appropriate no-flow temperature, the temperature at which the viscosity dramatically increases and induces the flow to stop, is the critical issue in these approaches [20]. In most cases, the volume filled by the melt is overestimated due to poor characterization of the material. For semicrystalline materials, a better knowledge of the viscosity-crystallization interplay could enhance the predictions of the replication accuracy by simulations.

In this paper, the replication of micro-features during the injection molding process has been evaluated by a two-step approach, in which the outputs of the simulation of the overall process (without considering the presence of micro-features) have been adopted as inputs for the simulation of micro-feature filling. A solidification criterion has also been proposed. A fully characterized material, also concerning the interplay between viscosity and crystallization, has been adopted. The results obtained by simulation, in terms of filling length into a micro-feature (i.e., the replication accuracy), have been compared with experimental results obtained through injection molding tests conducted under different conditions of cavity surface temperature.

## 2. Materials and Methods

An isotactic polypropylene (iPP, grade T30G, by Basell, Ferrara, Italy) was adopted for the injection molding tests. The viscosity and crystallinity interplay will be discussed in the following.

**Figure 1:**
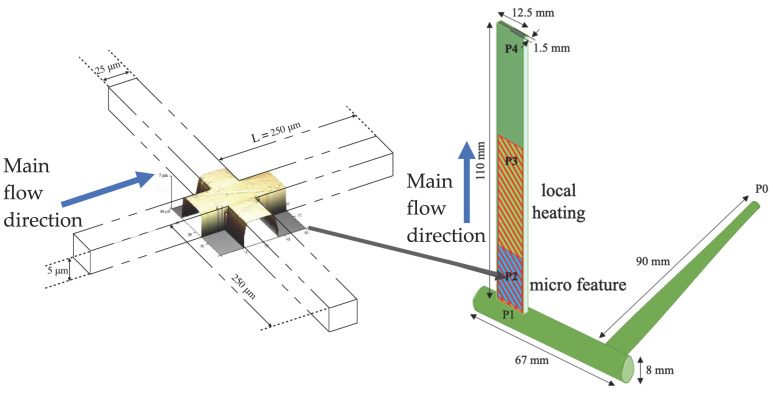
Sketch of the cavity geometry adopted for the injection molding test, with the position in which the insert with micro-crosses is located. The micro-cross AFM acquisition is also reported.

Injection molding was carried out with a fast cavity heating device. A rectangular cavity with a length of 110 mm, a width of 12.7 mm, and a thickness of 1.5 mm (as reported in Figure 1 was adopted for the experiments. A heating device, made of an electrically conductive layer of carbon black loaded poly(-imide-amide), was placed on both sides of the cavity, covering the first 70 mm of the cavity length downstream of the gate. A 19 mm × 19 mm nickel shim insert (described elsewhere [14]) with micro–features was placed on one of the cavity sides downstream of the gate (see Figure 1). The micro–features are composed of crosses with arms of rectangular cross-section. The crosses on the nickel insert are in relief and thus produce grooves on the surface of the molded samples. The length, L, of the four arms is 250 μm and the other nominal cross-section dimensions are: height of 5 μm and width of 20 μm.

A 70–ton Negri–Bossi reciprocating screw injection molding machine (Negri Bossi, Cologno Monzese, Milano, Italy) was adopted for producing the samples for the analysis of replicability. A volumetric flow rate of 2.8 cm^3^/s and an injection temperature of 220 °C were adopted during the tests. Holding pressure and time were 720 bar and 8 s, respectively. The whole mold was conditioned and kept at 27 °C. Pressure evolutions during the process were acquired in five positions along the flow path: P0 in the injection chamber, P1 in the runner, P2-P3-P4 inside the cavity, 15 mm, 60 mm, and 105 mm downstream from the gate position, respectively. Moreover, temperature evolutions were recorded at the cavity surface 20 mm downstream from the gate. Tests were conducted by supplying each heating device with electrical power densities of 7 and 10 W/cm^2^ (which means that the cavity surface temperature reaches, in position P2, 120 °C, and 150 °C, respectively) for a heating time of 13 s after the first contact with the melt. Moreover, some tests (defined in the following as Passive tests) were carried out without activating the heating devices.

Atomic Force Microscopy (AFM, Multimode Dimension V coupled with Nanoscope V, Veeco, Santa Barbara, CA, USA) was adopted for the analysis of the microfeature replication accuracy. OTESPA probe silicon cantilevers (Bruker, Billerica, MA, USA) with nominal radii of c.a. 7 nm and 300 kHz resonance frequency, were adopted for the replication detection. The free amplitude of the probe oscillation was 50 mV. A scan rate of 0.5 Hz and 256 points per line were selected for the acquisitions. Bruker NanoScope software (Bruker, Billerica, MA, US), version 7.30 was adopted for map acquisitions, and NanoScope Analysis software 1.8 (Bruker, Billerica, MA, US) was adopted to analyze the maps.

## 3. Injection Molding Modelling

As stated in the introduction, a multi-scale simulation approach was adopted to simulate the overall process of replication during the injection molding process. The first step was to simulate the filling and the packing stage in the macro-cavity (see Figure 1). The model adopted to simulate the process into the macro–cavity and the constitutive models are detailed in the supplementary materials section. The second step was to simulate the pressure driven flow into a micro cavity, the micro-corner in the cases reported in this work. Essentially, the replication process is simply the filling of the micro-corner due to a pressure driven flow.

**Figure 2:**
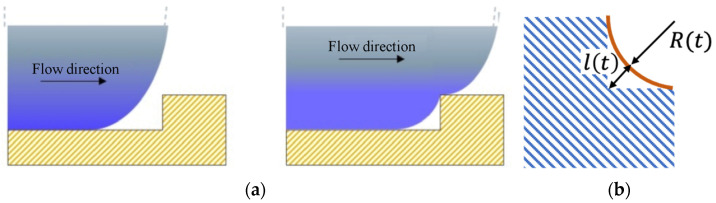
Schematic representation of the microstructure filling that occurs while the advancement of the flow front in the cavity proceeds (**a**). Geometrical representation of the polymer flow front during the microstructure filling (**b**).

A scheme to describe how the polymer fills the microstructure corner volume is shown in Figure 2a. When the melt advancing flow front contacts the micro cross, only a small part of the whole volume of the corner is filled by the polymer. The melt in contact with the cavity surface cools down to a temperature close to the cavity surface temperature. The formation of a frozen layer may occur, determining the end of the micro-cross filling. The filling of the micro-corner volume continues as long as the advancement of the flow front proceeds inside the cavity. The replication process is driven by the pressure at the cavity positions where the micro-corner is located, which build up not only during its first contact with the polymer but also during the subsequent steps of the process, namely the end of cavity filling and packing stages.

The microfeatures (the micro crosses in this work) replication quality can be influenced by the effects of both the counterpressure due to the trapped air and the capillary pressure.

The filling of the micro-corner can be sketched as shown in Figure 2b, where *l(t)* represents the distance between the polymer front and the unfilled edge. The hypothesis was that the filling proceeds keeping the flow front tangent to both the corned edges. As the filling of the micro-corner proceeds, the volume available for the air, namely the unfilled volume, decreases, and consequently, the pressure inside the air increases. Assuming isothermal conditions and considering the air as an ideal gas, air pressure evolution can be described as

(1) $$P_{air}\left(t\right)=\frac{V_{in}\cdot P_{in}}{V\left(t\right)}$$

where *P_in_* and *V_in_* are the initial air pressure and corner unfilled volume, respectively, *V(t)* is the unfilled volume at time t. Such a volume depends on the length *L* of the microstructure arm (see Figure 1) and *l*^2^*(t)*, which is a measure of the unfilled micro-corner (see Figure 2b). Thus, the air pressure evolves according to Equation (2).

(2) $$P_{air}\left(t\right)\propto \frac{V_{in}}{L\cdot l^{2}\left(t\right)}$$

According to the geometry shown in Figure 1, capillary pressure is given by Equation (3)

(3) $$P_{cap}\left(t\right)=\frac{\sigma }{R\left(t\right)}$$

where *R(t)* is the curvature of the melt front, σ is the surface tension that counteracts the pressure-driven flow. According to Rytka [21], the surface tension of iPP in mN/m can be calculated as a function of the temperature, *T*, expressed in °C adopting the expression

(4) σ=−0.12 T+41.2

The distance l(t) can be related to R as given in Equation (5):

(5) $$l\left(t\right)=R\left(t\right)\cdot \left(\sqrt{2}-1\right)$$

The dependency of $P_{cap}$ on the distance l(t) and the temperature can be obtained by replacing Equations (4) and (5) in the expression of $P_{cap}$ (Equation (3)):

(6) $$P_{cap}\left(t\right)=\left(\sqrt{2}-1\right)\frac{-0.12\cdot T+41.2}{l\left(t\right)}$$

**Figure 3:**
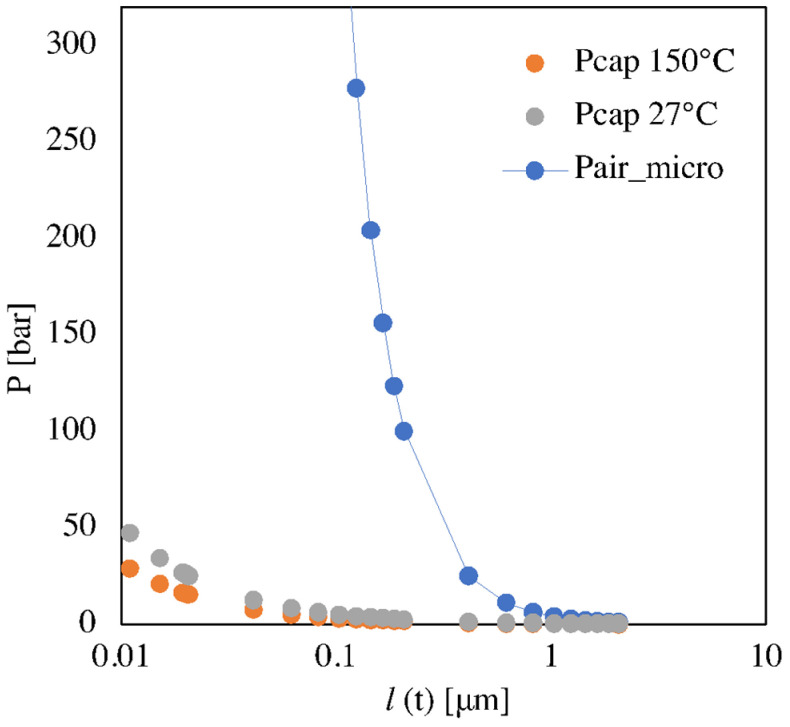
Capillary and trapped air pressure evaluated for microstructure with respect to the distance between the polymer front and the unfilled edge, namely *l(t)*.

Capillary pressure calculated by Equation (6) and the air pressure calculated by equation (2) are shown in Figure 3 versus *l(t)*. The capillary pressure starts to be significant for *l(t)* smaller than 0.2 μm. The effect of the temperature on the capillary pressure is negligible. Air pressure sharply increases as *l(t)* decreases. The capillary pressure is always smaller than air pressure. The performed analysis indicates that the capillary pressure can be neglected and the polymer flow during the micro-corner filling is mainly influenced by the air pressure increase.

**Figure 4:**
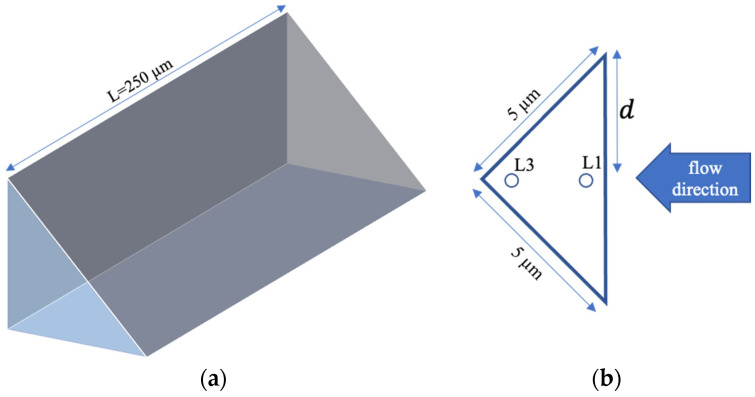
Schematic representation of the volume adopted in the simulations and equivalent to the microstructure corner (**a**). Section of the equivalent volume parallel to the flow direction (**b**).

The micro-corner was represented by a prism (as shown in Figure 4a) with a rectangular cross-section (Figure 4b). The prism thickness decreases along the flow direction (see Figure 4b, where the dimensions of the prism section parallel to the flow direction are also reported). The prism width L is constant and equal to 250 μm (see Figure 4a). The polymer advancing inside the micro-corner is driven by the pressure evolution in position P2, where the micro-corner is located. The filling of the micro-corner was simulated by imposing the pressure evolution evaluated in position P2 (which is essentially equal to the recorded pressure) as inlet pressure at the micro-corner entrance. To correctly describe the micro-corner filling, the effect of the trapped air pressure was accounted for in the software. Thus, the calculated pressure at the entrance of the corner, $P_{in}$, is given by Equation (7), where $\Delta P_{p}$ is the pressure drop required for the melt advancement.

(7) $$P_{in}\left(t\right)=\Delta P_{p}\left(t\right)+P_{air}\left(t\right)=P_{2}$$

At each time during the process, the volumetric flow rate was adjusted to obtain a pressure at the entrance, *P_in_*, equal to the pressure calculated in position P2, namely *P*_2_**.

## 4. Experimental Results

**Figure 5:**
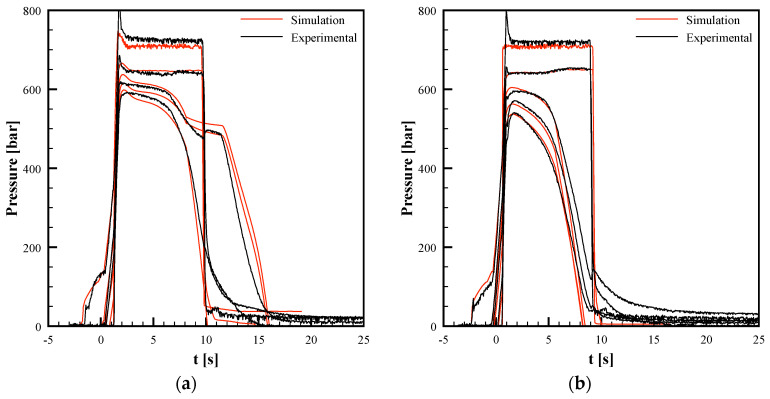
Recorded (black lines) and simulated (red lines) pressure evolutions during the injection molding tests for (**a**) the passive condition and (**b**) activating the heating devices with an electrical power density of 10 W/cm^2^ and heating time of 13 s.

Figure 5a,b shows the recorded pressure evolutions for the Passive test and for the test with an electrical power density of 10 W/cm^2^ (150 °C cavity surface temperature) and heating time of 13 s. Four positions along with the flow direction were considered: the injection position, P0, the sprue, P1, 15 mm downstream from the cavity entrance, P2, 60 mm from the cavity entrance, P3, and the end of the cavity, P4. The time t = 0 corresponds to the time at which the melt reaches position P2, where the micro–features are located; the injection starts 2 s in advance with respect to the first contact in P2, namely at t = −2 s in the plots of Figure 5. When the polymer completely fills the cavity (at about 0.8 s), the pressure in all the positions undergoes a sharp increase. During the packing step, the pressures remain essentially constant in positions P0 and P1. The pressure exerted by the screw on the polymer generates a packing flow that compensates for the polymer shrinkage inside the cavity due to both the cooling (determined by the contact of the melt with the cavity surface) and the resultant crystallization. When the heating devices are powered (see Figure 5b), the temperatures increase and crystallization slows down (or disappears); consequently, the pressure drop between P2 and P3 decreases. During packing, the pressure-drop across the gate, namely between positions P1 and P2, grows following the gate solidification. Starting from that time (namely about 8 s), additional material cannot enter the cavity and the pressure drop between P0 and P1 slightly decreases due to the packing flow intensity reduction. For the Passive test, after the gate sealing time (t = 6 s, see Figure 5a), recorded pressures in all the cavity positions decrease with a constant rate. If the heating devices are activated for times longer than the gate sealing time (see Figure 5b), pressures in positions P2 and P3 go to zero in two different stages: during the first step down, pressures reach intermediate (500 bar) after gate sealing, at the heating device deactivation, pressures decrease faster to zero.

**Figure 6:**
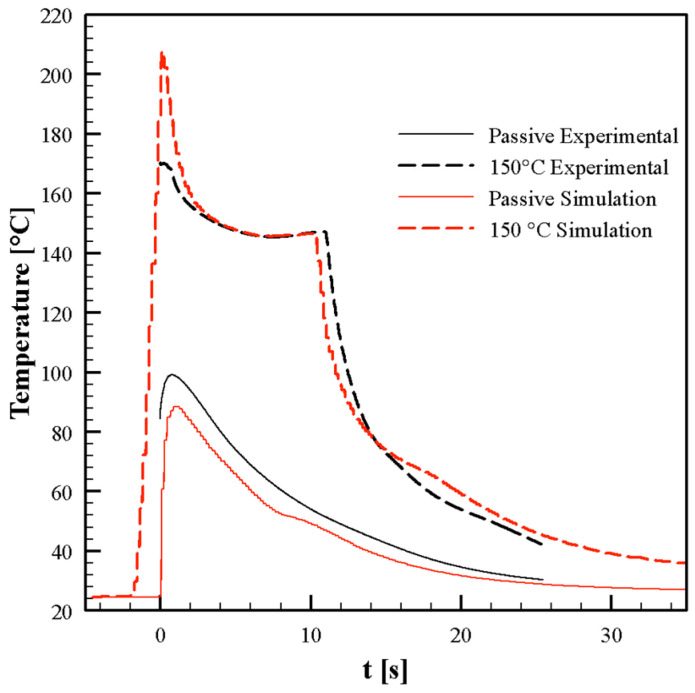
Recorded (black lines) and simulated (red lines) temperature evolutions in position P2 during the injection molding tests for the passive condition (solid lines) and activating the heating devices with an electrical power density of 10 W/cm^2^ and heating time of 13 s (dashed lines).

Figure 6 shows measured temperature evolutions in the Passive test and for the test with an electrical power density of 10 W/cm^2^ and heating times of 13 s, whose pressure evolutions are reported in Figure 5. Furthermore, for these graphs, t = 0 s corresponds to the time at which the polymer reaches position P2. Temperature sharply increases before the melt reaches P2, when the heating device is activated, up to a maximum due to the contact of the hot melt with the cavity surface. After that, temperature decreases toward the asymptotic temperature that depends on the electrical power density adopted: with an electrical power density of 10 W/cm^2^ an asymptotic temperature, T_a_, of 150 °C was achieved 6 s after the heater activation. At the deactivation of the heating device, temperature decreases down to the mold temperature, namely 27 °C.

**Figure 7:**
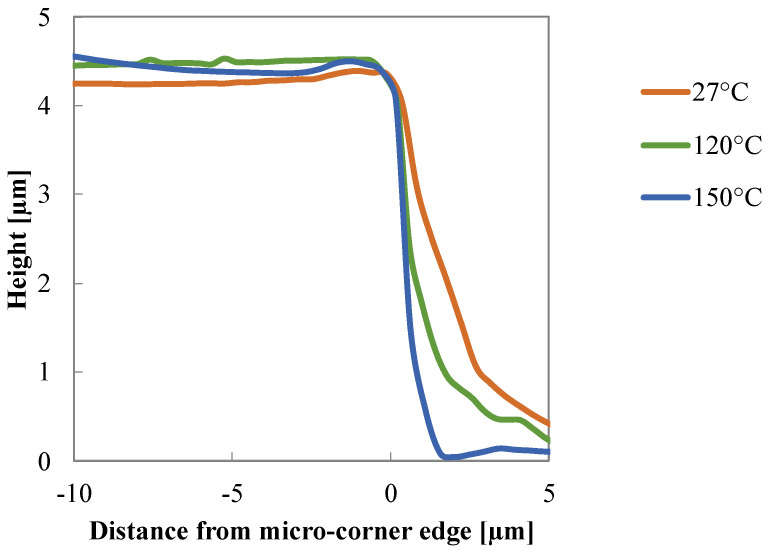
AFM height profiles acquired on the crosses obtained in the molded samples.

AFM was adopted to investigate the replication quality of the micro–features. As mentioned above, the crosses on the nickel insert produce grooves on the surface of the molded samples. AFM height maps were acquired on several samples obtained, adopting different values of electrical power density (which means different T_a_) and different heating times. Analysis of the AFM patterns revealed that, according to the literature [22,23], replication quality improves when higher temperatures of the cavity surface are adopted, the effect of the heating time is almost negligible. Therefore, in the following only AFM acquisitions on the molded samples obtained with a heating time of 13 s will be considered. AFM acquisitions are affected by the so-called convolution effect, due to the interaction between sample and AFM probe. This effect becomes significant during the acquisition of structures with sharp details, such as in the proximity of undercuts [24]. Some methods were adopted in the literature, and in this work, to reduce the effect of sample–probe interaction [25]. Figure 7 shows the height profiles acquired on the molded samples where micro-features are located (position P2 along with the flow direction). The increase of the cavity surface temperatures enhances the replication accuracy: the filling depth of the melt inside the micro-feature, the corner of the crosses in these cases, increases. With a 27 °C cavity surface temperature, only a short filling length is achieved. The increase of the cavity surface temperature up to 120 °C drives a significant increase of the filling length. The volume can be considered completely filled by the polymer when a cavity surface temperature of 150 °C is adopted. The height profile of the micro-feature located inside the cavity coincides with the height profile obtained on the sample produced with 150 °C cavity surface temperature, therefore, the volume filled by the polymer is 100%.

Table 1 summarizes the effect of the cavity temperature on the percentage (the volume of the corned filled by the polymer with respect to the volume of the corner).

Numerical simulations were conducted under two conditions at the cavity surface: 27 °C for the whole process, and 150 °C cavity surface temperature held for 13 s. Figure 6 shows the comparison between the experimental and the simulated temperature evolutions at cavity surface in position P2. In both cases, the main features of the experimental temperature evolutions are correctly described by the numerical simulations. Predictions underestimated the experimental temperature peak, especially when the heater was activated. Figure 5a,b show the comparisons between the experimental and the simulated pressure evolutions in positions P0–P4, for all molding conditions considered in this work. Predictions are consistent with the experimental pressure histories. In particular, the effects of the cavity surface temperature evolution on the gate sealing time (which determines a small pressure increase in P1) were nicely described by predictions. The agreement between the experimental and simulated results concerning temperature and pressure evolutions confirm and validate the proposed model and the material characterization adopted. Once UNISA software was validated, the filling code was adopted to describe the filling of the micro-corner. As shown in the previous paragraph, the filling of the micro-corner occurs while the melt completes the filling of the macro cavity, and also during the packing stage.

## 5. Discussion

Simulations of injection molding tests, in Passive conditions (without any heating of the cavity surface), and conditions with a 150 °C cavity surface temperature held for 13 s were conducted. Figure 7 shows that during experiments, the micro-corner volume was only partially filled in the Passive conditions, whereas it was completely filled in the conditions with 150 °C cavity surface temperature. Vice-versa, simulations predicted that the corner volume is completely filled in both conditions.

### 5.1. Simulation of Micro–Corner Filling

**Figure 8:**
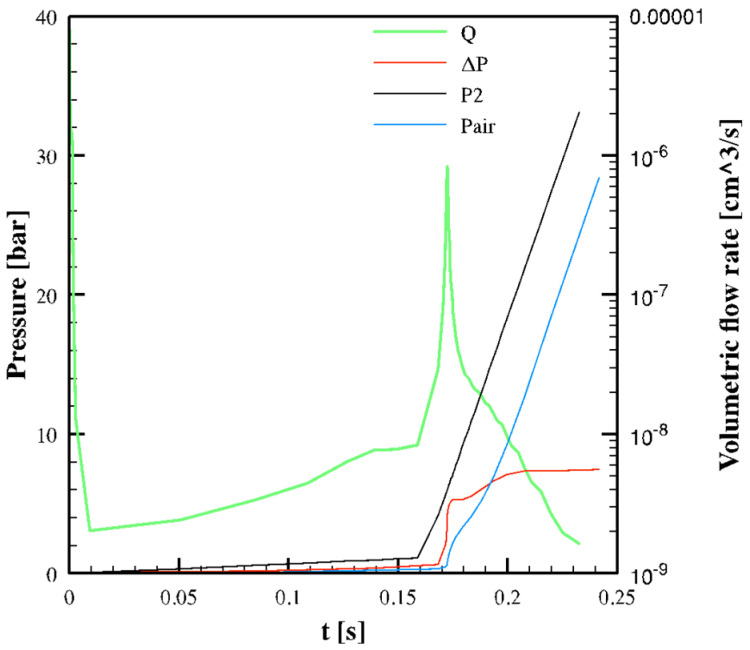
Evolution of pressures and volumetric flow rate in the micro-corner calculated for Passive condition.

Figure 8 shows the evolution of pressures and volumetric flow rate in the micro-corner for the Passive condition. A sharp increase in the volumetric flow rate is calculated when the imposed pressure *P*_2_ shows a slope change. Air pressure increases as the filled volume increases. As soon as *P_air_* becomes significant, volumetric flow rate decreases. The pressure-drop Δ*P_p_* in the polymer flowing into the micro-corner remains essentially constant, and it is largely overcome by the air pressure. The micro-corner volume is filled in 0.242 s.

**Figure 9:**
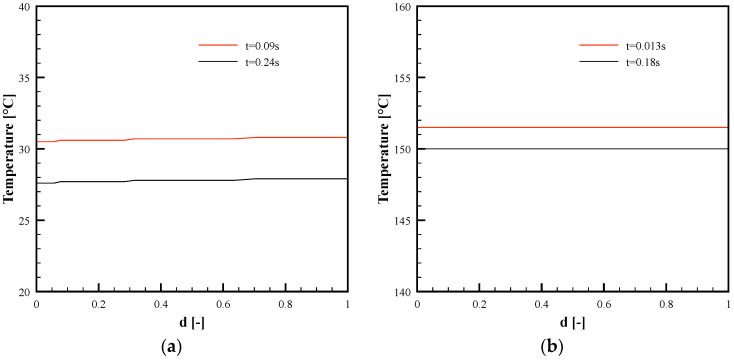
Temperature distributions along the dimensionless half thickness from the corner surface, d, in positions L1 and L3 for Passive condition (**a**) and 150 °C cavity surface temperature condition (**b**).

Figure 9 shows the predicted temperature distribution along the local dimensionless half thickness, *d·*(*d* = 1)corresponds to the micro-corner midplane), in two positions, L1 and L3 (see Figure 4b), for both cases, 27 °C and 150 °C cavity surface temperature. In particular, Figure 9 shows the temperature distributions at two times from the beginning of the micro-corner filling: when the polymer reaches position L1 (shown in Figure 4b), and the end of the micro-corner, position L3. Obviously, the times are different for the two selected conditions: when 25 °C was adopted as cavity surface temperature, the melt reaches the positions in longer time than the case in which 150 °C was adopted. Micro-corner filling occurs in quasi–isothermal conditions, nearly at the cavity surface temperature.

**Figure 10:**
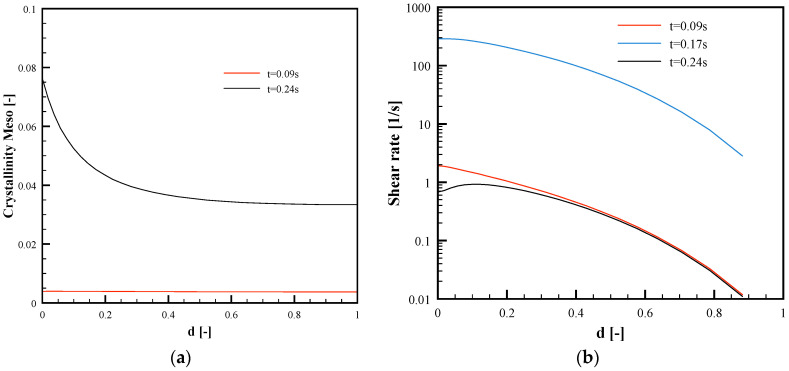
Mesophase crystallinity (**a**) and shear rate (**b**) distributions along the dimensionless half thickness, *d*, in position L1 for the Passive condition.

Figure 10a shows the mesophase crystallinity distribution along d in position L1 at the same times analyzed in Figure 9a, for the Passive case. The formation of the sole meso phase was predicted, with amount increases with time, although a low amount, below 10%, was achieved. The alpha phase was almost absent. For the test with a 150 °C cavity surface temperature, crystallization did not occur during the micro-corner filling.

Figure 10b shows the shear rate distributions for the Passive condition in three different time steps: the time at which the melt reaches position L1 (t = 0.09 s), the time at which the melt reaches the micro-corner tip (t = 0.24 s), and the time at which the flow rate shows a maximum (t = 0.17 s, see Figure 8). Shear rate decreases from the surface to micro-corner midplane, where symmetry condition is imposed. At t = 0.17 s, the shear rate assumes significantly high values, in the order of hundreds of s^−1^.

The injection molding process is carried out with a cycle time that is comparable with the crystallization time, and the solidification process proceeds from the sample surface to the midplane due to the material cooling and/or crystallization. Polymer crystallizes close to the surface, and this causes the formation of a layer with very low shear rate, a layer which progressively grows during the process. In the case of the macro-cavity filling, the UNISA software correctly predicts the material solidification during the process. However, in the case of the micro-corner filling, the numerical simulation is not able to predict the hinder of filling due to the solidification of the material (as experimentally observed); as a result, at a 27 °C cavity temperature, the micro-corner is completely filled.

### 5.2. Enhancement of Micro–Corner Filling Simulation: Viscosity Modification

**Figure 11:**
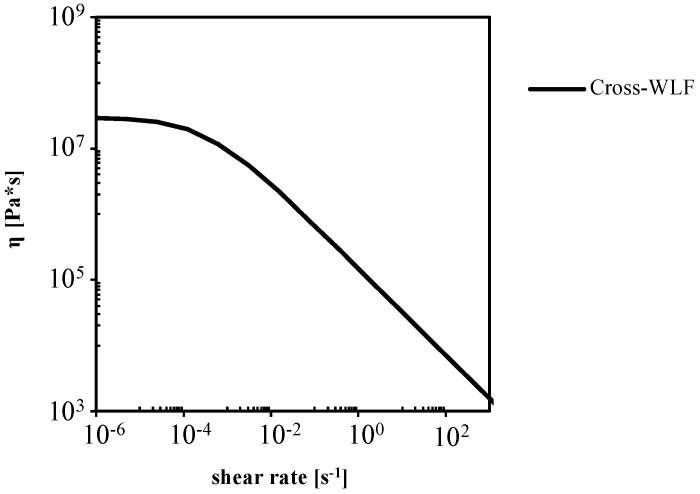
The T30G flow curve was calculated from the Cross-WLF model at 27 °C and in absence of crystallization.

Figure 11 shows the T30G flow curve calculated from the Cross-WLF model at 27 °C, in absence of crystallization. The shaded area in Figure 11 identifies the range of shear rates experienced by T30G during the micro-corner filling, it corresponds to the power-law zone of the flow curve. In that zone, viscosity decreases as the shear rate increases: the viscosity is not sufficiently high to significantly hinder polymer flow in absence of crystallization.

**Figure 12:**
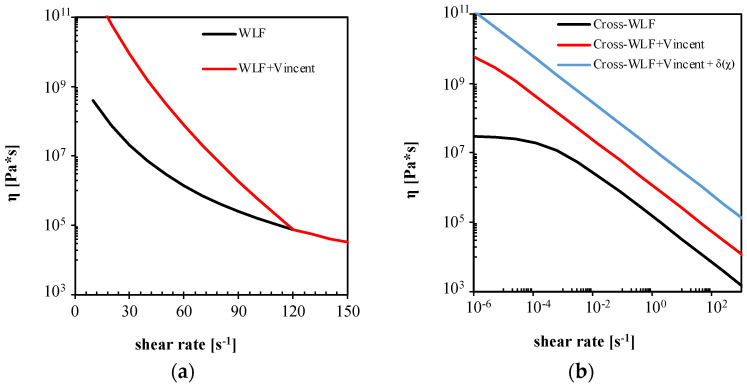
Modification of η_0_ introduced by Vincent according to Equation (8) (**a**). Effect on T30G viscosity flow curve of the modification proposed by Vincent and considering a total crystallinity of 20% (**b**).

The parameters of the Cross-WLF model, describing the dependence of viscosity on pressure and temperature, were defined through rheological measurements performed at high temperatures, far above 27 °C. This can induce a poor description of the rheological behavior at temperatures significantly lower (in the order of 100 °C lower) than those adopted for defining the rheological parameters. Thus, the material flows also at low temperatures, in conditions of very fast cooling, if it has not had sufficient time for crystallization. The parameters usually adopted for Cross-WLS are not suitable to describe the rheological behavior under the cooling rates in the order of 100 °C/s, as during the micro-corner filling. To overcome this limitation, Vincent et al. [17] proposed, for a different grade of isotactic polypropylene, a modification of the WLF model, as given in Equation (8):

(8) $$\eta_{0}=\eta_{0}^{WLF}\left(P,\;T,\;\chi \right)\;if\;T\;\geq \;T_{s}\;\eta_{0}=\eta_{0}^{WLF}\left(P,\;T,\;\chi \right)e^{b\left(T_{s}-T\right)}\;if\;T\;<\;T_{s}$$

where *T_s_* and b are material constant. The modification introduced by Vincent consists of a viscosity increase by the effect of an additional exponential factor, starting below *T_s_* and growing with *T_s_ − T*. The effect of the proposed modification on the rheological model when *T_s_* = 120 °C and *b* = 0.1 is shown in Figure 12.

As shown by the red line in Figure 12a, below 120 °C, $\eta_{0}$ increases as temperature decreases; at 27 °C the Newtonian viscosity increase is three orders of magnitude higher that the viscosity calculated by the unmodified Cross-WLF model. Figure 12b shows that the proposed modification causes a viscosity increase for all shear rates at 27 °C. The viscosity increase from the effect of Vincent’s modification reduces in the power-law region. In particular, the viscosity increases by only one order of magnitude than in the Newtonian region.

**Figure 13:**
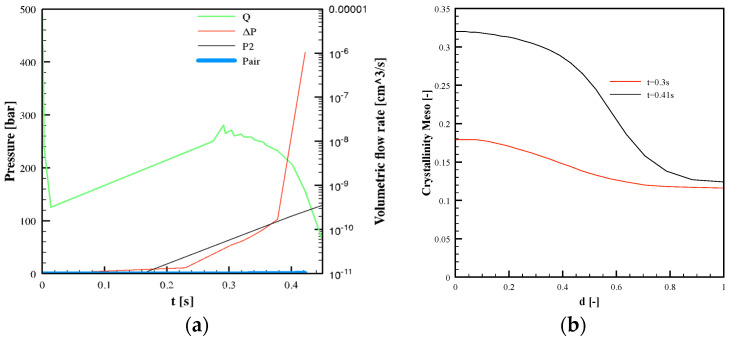
Evolution of pressures and volumetric flow rate in the microstructure corner (**a**) and overall crystallinity distributions along the dimensionless distance from the corner surface, d, in position L1 (**b**). In both plots are shown variables calculated for the Passive condition by adopting Vincent’s viscosity modification.

The modified viscosity model (Equation (8)) was implemented in the UNISA software to perform simulations of the micro-corner filling during molding tests. The simulation of the whole injection molding process was conducted, and it was verified that Vincent’s modification of viscosity does not influence the main features of the process, the gate sealing time, and the solidification/crystallization process. Concerning the micro-corner filling, the Passive conditions show a reduction of the micro-corner filled volume, whereas the case with a 150 °C cavity surface temperature is almost unaffected by Vincent’s modification of viscosity. Figure 13a shows the evolution of pressures and volumetric flow rate for the Passive condition.

**Figure 14:**
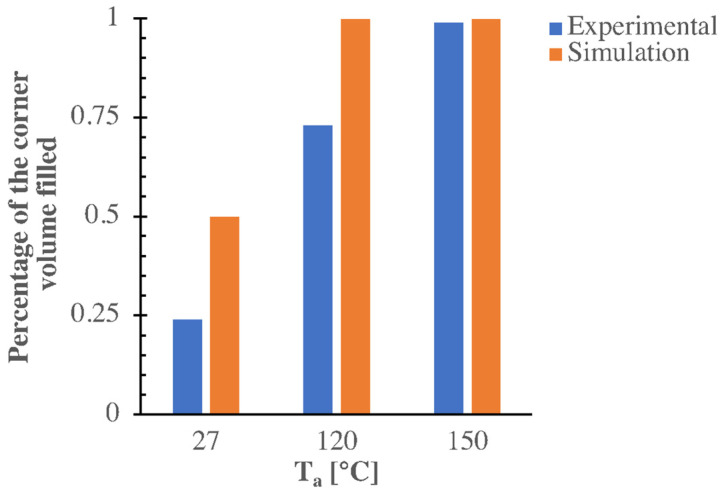
Experimental and calculated percentage of the corner volume filled for the three molding conditions considered in this work.

Figure 13a shows that the volumetric flow rate almost linearly increases with the imposed pressure *P*_2_. Moreover, the volumetric flow rate values are smaller than those calculated in simulations without Vincent’s modification of viscosity, due to the higher values of viscosity. This means that the advancing front slows down, and as a result, the micro-corner volume is only partially filled. Furthermore, such a slowdown allows an increased degree of crystallization, mostly referred to as the meso phase, which contribute to hindering the micro-corner filling. In position L1, high values of crystallinity are predicted along the corner half-thickness (see Figure 13b). In particular, close to the sample surface, crystallinity is about 30% at t = 0.4 s. The presence of high crystallinity amounts induces a further increase in viscosity, leading to polymer solidification. Figure 12b also shows the flow curve calculated from Vincent’s model considering the effect of crystallinity for χ = 20%. The increase in crystallinity also leads to a significant increase in viscosity in the power-law zone. This explains the sharp increase in the pressure drop in the polymer shown in Figure 13a at about t = 0.4 s; the pressure drop largely overcomes the pressure imposed at the corner entrance, thus, in the corner, the polymer flow stops. Figure 14 shows the results of the filling corner simulations obtained for all the three cases proposed in this work.

The simulations consistently reproduce the experimental results for the cases with 27 °C and 150 °C cavity surface temperatures. In particular, with 27 °C the viscosity increase from Vincent’s modification induces a slowdown in the melt advancement inside the micro–corner. This leads to crystallization into the mesomorphic phase, which, in turn, causes polymer solidification and stop. The case at 120 °C shows a complete filling of the corner, although the experimental results showed a smaller filled volume. This discrepancy could be due to the fact that the parameters of Vincent’s model are not optimized for the polypropylene grade [17] and for the processing conditions adopted in this work. Rheological characterization in the shear flow of the polymeric material is usually performed at temperatures well above the solidification. The rheological material parameters could be very different from those obtained by conducting the aforementioned rheological experiments. The discrepancy found at the lowest temperatures (Figure 14) could be due to fact that the molding tests were conducted with mold temperatures well below those adopted for iPP characterization. This requires further investigation of iPP rheological behaviour at those temperatures.

Furthermore, as the micro-corner is a convergent cavity (see sketch showed in Figure 4), the extensional flow should also be considered. The extensional flow is recognized to be more efficient than the shear flow in orienting molecules, thus enhancing the crystallization kinetics [26], leads to a significant increase in viscosity. This phenomenon, during micro–corner filling, induces premature polymer stop, and a better description of corner volume filling.

## 6. Conclusions

The evolution of cavity surface temperature during the injection molding process allows surface properties of the molded parts to be tailored, thanks to the replication of micro and nanostructures present on the cavity surface. The possibility of replicating micro and nanostructures is related to the control of cavity temperature during the process; experimental results on an isotactic polypropylene confirmed that the replication accuracy is enhanced as the cavity surface temperature increases. The presence of a cavity characterized by macro-metrical dimensions, with a thickness in the order of few millimeters, and the features to be replicated in the order of few micros, is the main challenge in the simulation of the injection molding process. For this reason, a multiscale step approach was adopted in this work to simulate the overall process. The first step consisted of simulating the injection molding into a microcavity, without considering the presence of the micro-features. The second step adopted the output of the first step to simulate the replication process, considered as a pressure-driven flow into a micro-cavity, a micro-corner in these cases. Such a pressure driven flow accounts for the presence of trapped air which compresses due to the entrance of the melt. In a first stage, the rheological model, based on the Cross-WLF equation, for the isotactic polypropylene adopted for both molding tests and simulations, did not allow for a reliable description of the micro-corner filling, the filling was complete with very low cavity surface temperature, i.e., 27 °C. A modification of the model adopted for describing rheological behavior was adopted for simulating both the filling into the macro-cavity and the replication process. Such a modification introduced a further increase in viscosity for temperatures below those considered during rheological tests, which are close to the crystallization temperature. At 27 °C, where the mesomorphic kinetics is predominant, the melt slowdown induced by the viscosity increase gives sufficient time for mesomorphic phase formation; in turn, the incoming crystallization induces a further viscosity increase that causes the polymer stop consistently with experimental observation. The simulation of the macro-cavity filling gives results, in terms of pressure and temperature evolutions, consistent with the expectation, in terms of polymer solidification. Moreover, the replication accuracy increases with the cavity temperature. However, the accuracy of the replication is overestimated at low temperatures, probably due to the lack of description of the crystallization kinetics under high orientation in the presence of strong flow (shear and/or elongation), which forms close to the cavity walls.

## Figures and Tables

**Table 1:** Percentage of the corner volume filled by the polymer for the different molding conditions adopted.

Cavity Surface Temperature, T_a_ [°C]	Percentage of the Corner Volume Filled
27 (Passive)	24%
120	73%
150	100%

## Supplementary Materials

The following are available online at https://www.mdpi.com/article/10.3390/polym13193236/s1, References [27,28,29] are cited in the supplementary materials.
