# Supplementary material for: Multi-Scale Simulation of Injection Molding Process with Micro–Features Replication: Relevance of Rheological Behaviour and Crystallization
Source: Polymers (Basel). 2021 Sep 24;13(19):3236. doi: 10.3390/polym13193236 (PMC8512435; doi:10.3390/polym13193236)
Supplement: Supplementary file 1 [file polymers-13-03236-s001.zip › polymers-1318215-supplementary.pdf]

Modelling of filling and packing stages into the macro-cavity

In this work, the UNISA software, already developed for the simulation of the injection molding process, was modified and updated to perform numerical simulations of the micro-corner filling,. The UNISA software describes the polymer flow from the injection chamber to the cavity by considering a series of rectangular or cylindrical elements. The transport equations are solved adopting the lubrication approximation, namely, one–dimension laminar flow of a viscous non–Newtonian fluid in non–isothermal conditions.

Under the assumption of lubrication, the predominant velocity is those along the flow direction (x coordinate), which depends on the thickness coordinate (y coordinate). The momentum balance becomes:

$$\frac{d\tau_{yx}}{dy} = -\frac{dP}{dx} \tag{1}$$

where τ_{yx} is the stress acting along with the flow directio (x) on a surface of normal y, and P is the pressure. Under the hypothesis that pressure is constant along the thickness direction, the velocity is given by equation 2:

$$u(x) = -\frac{dP}{dx}\int_{y}^{H(x)}\frac{y}{\eta}\,dy \tag{2}$$

where η is the viscosity, and $H(x)$ is the cavity thickness. Thus, combining equation 1 and 2, it is possible to obtain the volumetric flow rate Q, whatever x:

$$Q = -2W\frac{dP}{dx}\int_{0}^{H(x)}\int_{y}^{H(x)}\frac{\alpha}{\eta}\,d\alpha\,dy \tag{3}$$

where W is the cavity width. Equation 3 gives the relationship between the pressure gradient and the flow rate for a viscous fluid at any temperature and velocity gradient distribution. Symmetry conditions were assumed at the cavity midplane, and no-wall slip condition at the cavity surface.

In the heat transfer equation, the convective term along the flow direction, the conductive term along with the sample thickness, the crystallization latent heat, and the viscous generation are considered. The energy balance is given by equation 4:

$$\rho\,Cp\left(\frac{\partial T}{\partial t} + u\frac{\partial T}{\partial x}\right) = k\frac{\partial^2 T}{\partial y^2} + y\frac{\partial P}{\partial x}\frac{\partial u}{\partial y} + \lambda\rho\frac{\partial \chi}{\partial t} \tag{4}$$

where ρ is the material density, assumed constant during the filling stage, Cp is the specific heat, k is the thermal conductibility, λ is the latent heat of crystallization, χ is the crystallinity degree.

Symmetry conditions were applied at the midplane and a heat transfer coefficient, depending upon the local contact time of the melt with the mold, was considered at the surface. Additional assumptions are made during the filling, packing, and cooling stages. During the filling stage, the material is considered incompressible, and the flow rate is imposed as an inlet condition. The fountain flow is not implemented but the variables at the flow front are averaged based on flow rate (i. e. cup-mixing variables are considered at the front). During the packing stage, the material is considered compressible and the flow rate, at each axial position, is determined by the downstream densification. The mass flow rate will be given by equation 5, where L is the cavity lenght:

$$\dot{M}(x,t) = 2W\int_{x}^{L}\int_{0}^{H(x)/2}\frac{\partial\rho(P,T,\chi)}{\partial t}\,dy\,d\alpha \tag{5}$$

During the cooling stage, the pressure evolution is evaluated based on the PVT behavior of the material, accounting for crystallization kinetics. The effect of mold deformation, due to the polymer pressure, is accounted for during the packing and cooling stages. The complete/extensive characterization of the iPP considered in this work allowed including models in the software to describe rheology, crystallization kinetics, volumetric behavior, and their mutual effects. A multi-phases crystallization kinetics model considering meso and alpha phase evolution is considered. A flow-induced crystallization model, based on the

molecular stretch, is considered for the alpha phase morphology evolution [1]. Molecular stretch evolution is described adopting a non-linear Maxwell's model with a single relaxation time, under constant temperature and pressure, dependent upon the molecular stretch [2]. Density and viscosity are considered dependent also upon crystallinity. Material solidification is considered a gradual phenomenon, described by the viscosity increase on temperature decrease, and both crystallinity and pressure increases. The iPP viscosity was described by a modified Cross model accounting for the crystallization evolution, as given in equation 6.

$$\eta(T,P,\dot{\gamma},\chi) = \frac{\eta_0(T,P)\cdot\delta(\chi)}{1+\left(\frac{\eta_0(T,P)\cdot\delta(\chi)\cdot\dot{\gamma}}{\tau_R}\right)^{1-n}} \tag{6}$$

where n and τ_R are material parameters. Equation 6 gives, for low values of shear rates, a constant Newtonian viscosity, whereas a power-law behavior is obtained at high shear rates. The Newtonian viscosity η_0 was described by the well–known WLF equation

$$\eta_0(T,P) = D_1 \cdot 10^{\frac{-A_1(T-D_2-D_3P)}{A_2+T-D_2}} \tag{7}$$

which describes the Newtonian viscosity dependence upon pressure and temperature, whereas the factor $\delta(\chi)$ in equation 6 describes the effect of crystallinity, χ, on viscosity according to the relationship given in equation 8 [1,3].

$$\delta(\chi) = e^{h\cdot\chi^2} \tag{8}$$

At each temperature, the effect of crystallinity on the viscosity is not constant along the flow curve. At low shear rates, the viscosity increase is of the order of $\delta(\chi)$ whereas at high shear rates it is the order of $\delta(\chi)^n$, and n is smaller than 1 for most of semicrystalline polymers.

The description of the evolution of the undirsturbed volume during the crystallization process is given by the Nakamura's approach, which concerns the crystallization toward the mesomorphic phase, and the Kolmogoff's approach, which concerns the evolution toward the alfa phase. In the model adopted for crystallization kinetics, the two phases compete for the same available amorphous part, and the evolution of each crystalline phase is given by

$$\frac{d\xi_i}{dt} = (1-\xi)\frac{dk_i}{dt} \tag{9}$$

where ξ is the overall relative crystalline degree, ξ_i is the relative crystalline degree of the i-phase, k_i is the evolution of the undisturbed volume of the i-phase. Kolmoroff's equation describes the evolution of the undisturbed volume of the α-phase:

$$k_\alpha = \frac{4}{3}\pi\int_0^t \frac{dN(s)}{dt}\left[\int_s^t G(u)du\right]^3 ds \tag{10}$$

Where N and G are the nucleation density and the growth rate, respectively. They are given by equations 11 and 12:

$$N(T(t)) = \frac{N_0}{1+A_n e^{B_n(T-T_m)}} \tag{11}$$

$$G(T(t)) = G_0 e^{-\frac{U}{R(T-T_\infty)}} e^{-\frac{k_g(T+T_m)}{2T^2(T_m-T)}} \tag{12}$$

where $T_m = T_{m,0} + \alpha_m P$ and $T_\infty = T_{\infty,0} + \alpha_\infty P$

Nakamura's equation describes the evolution toward the mesomorphic phase:

$$k_m = ln2\left[\int_0^t K(s)ds\right]^3 \tag{13}$$

where K is given by equation 14:

$$K(T(t)) = K_0 e^{-4ln2\frac{(T-T_{max})^2}{D^2}} \quad (14)$$

All the parameters are given in Table 1.

At 140 °C, with a 20% crystallinity degree, in the Nwwtonian zone, the viscosity increases by three orders of magnitude with respect to the viscosity evaluated without considering crystallization, whereas, in the high shear rates power-law zone, the viscosity increase reduces only to one order of magnitude. Table 1 summarizes the parameters adopted for equations 6-14.

Table S1. Parameters adopted for T30G in the constitutive models described by equations 6-14

Parameter	Value	Equation
n [–]	0.34	6
τ_R [Pa]	9850	6
D_1 [Pa s]	7664	7
A_1 [–]	1.74	7
D_2 [K]	503	7
D_3 [K/bar]	0.18	7
A_2 [K]	301.4	7
h [–]	180	8
N_0 [nuclei cm^{-3}]	1.95 10^9	11
A_n [-]	1.30 10^8	11
B_n [K^{-1}]	0.155	11
G_0 [cm s^{-1}]	1380	12
U/R [K]	751.6	12
K_g [K^2]	371381	12
$T_{m,0}$ [K]	467.54	11,12
α_m [K bar^{-1}]	0.01643	11,12
$T_{\infty,0}$ [K]	198.46	12
α_∞ [K bar^{-1}]	0.03129	12
K_0 [s^{-1}]	3.12	14
$T_{max,0}$ [K]	297.48	14
D [K]	29.95	14
α_{max} [K bar^{-1}]	0.04578	14

References

1. Pantani, R.; Speranza, V.; Titomanlio, G. Effect of flow-induced crystallization on the distribution of spherulite dimensions along cross section of injection molded parts. *Eur. Polym. J.* **2017**, *97*, 220–229.
2. Speranza, V.; Liparoti, S.; Pantani, R.; Titomanlio, G. Prediction of morphology development within micro–injection molding samples. *Polymer (Guildf).* **2021**, 123850.
3. Pantani, R.; De Meo, A.; Speranza, V.; Titomanlio, G. Effect of crystallinity on the viscosity of an isotactic polypropylene.; 2015; p. 020065.
